# Influence of Flavors on the Propagation of E-Cigarette–Related Information: Social Media Study

**DOI:** 10.2196/publichealth.7998

**Published:** 2018-03-23

**Authors:** Jiaqi Zhou, Qingpeng Zhang, Daniel Dajun Zeng, Kwok Leung Tsui

**Affiliations:** ^1^ Department of Systems Engineering and Engineering Management City University of Hong Kong Kowloon Tong China (Hong Kong); ^2^ Shenzhen Research Institute City University of Hong Kong Shenzhen China; ^3^ Department of Management Information Systems Eller College of Management The University of Arizona Tucson, AZ United States; ^4^ State Key Laboratory of Management and Control for Complex Systems Institute of Automation Chinese Academy of Sciences Beijing China

**Keywords:** e-cigarettes, flavors, social media, information propagation, social networks, electronic nicotine delivery systems, flavoring agents, information dissemination, social networking

## Abstract

**Background:**

Modeling the influence of e-cigarette flavors on information propagation could provide quantitative policy decision support concerning smoking initiation and contagion, as well as e-cigarette regulations.

**Objective:**

The objective of this study was to characterize the influence of flavors on e-cigarette–related information propagation on social media.

**Methods:**

We collected a comprehensive dataset of e-cigarette–related discussions from public Pages on Facebook. We identified 11 categories of flavors based on commonly used categorizations. Each post’s frequency of being shared served as a proxy measure of information propagation. We evaluated a set of regression models and chose the hurdle negative binomial model to characterize the influence of different flavors and nonflavor control variables on e-cigarette–related information propagation.

**Results:**

We found that 5 flavors (sweet, dessert & bakery, fruits, herbs & spices, and tobacco) had significantly negative influences on e-cigarette–related information propagation, indicating the users’ tendency not to share posts related to these flavors. We did not find a positive significance of any flavors, which is contradictory to previous research. In addition, we found that a set of nonflavor–related factors were associated with information propagation.

**Conclusions:**

Mentions of flavors in posts did not enhance the popularity of e-cigarette–related information. Certain flavors could even have reduced the popularity of information, indicating users’ lack of interest in flavors. Promoting e-cigarette–related information with mention of flavors is not an effective marketing approach. This study implies the potential concern of users about flavorings and suggests a need to regulate the use of flavorings in e-cigarettes.

## Introduction

The electronic cigarette, commonly known as an e-cigarette or electronic nicotine delivery system, is a method of delivering vaporized nicotine instead of combusting tobaccos. The advent of e-cigarettes provided smokers with an alternative way to give them a feeling similar to smoking but with less smoke ingestion, which is the major danger from using conventional cigarettes. A series of studies revealed the increasing popularity and ever-use of e-cigarettes in developed countries (eg, United Kingdom, United States), particularly among adolescents and young adults [1-4]. During 2011 to 2015, the use of e-cigarettes among high school students increased from 1.5% to 16.0% in the United States [4]. Today, more than 2.7 million people have used e-cigarettes worldwide. Sales reached more than US $2.8 billion in 2015 alone [5].

E-cigarettes are known to be effective in smoking cessation and less harmful in terms of the level of toxicants ingested [6-11]. Flavors of e-cigarettes play a critical role in enhancing the experience for e-cigarette users and helping smoking abstinence [12]. Thus, promoting flavors has become a major marketing strategy for e-cigarette manufacturers and retailers [13]. However, e-cigarettes may be harmful, as they could attract nonsmokers or former smokers to use conventional cigarettes [14]. The addition of flavors introduces new health risks to the use of e-cigarettes. Biochemical research identified positive correlations between cytotoxicity and the use of chemicals in flavor fluids [15-17]. In addition, similar to conventional tobacco products, the use of flavors in e-cigarettes is appealing to youth, young adults, and even children [3,18,19].

Despite the wide adoption and potential risks associated with e-cigarettes, regulation and legislation pertaining to e-cigarettes are still at their nascent stage. Researchers found an association between the popularity of e-cigarettes and stronger tobacco control, indicating that e-cigarettes are used to bypass smoking restrictions [1]. The US Food and Drug Administration has raised the concern that certain flavors’ appeal to young adults could lead to their initiating smoking [20]. Due to the lack of appropriate restrictions, the excessive level of flavor chemicals in e-cigarettes might irritate the respiratory system [18,21]. Regulatory authorities and policy makers are urged to learn more about e-cigarettes and their flavors, particularly from e-cigarette users [3,12,13,16,20,22].

Social media provides valuable resources for studying e-cigarettes. Social media users have formed online communities to discuss various topics relating to e-cigarettes, such as their flavors, use of e-cigarettes in smoking cessation, and the safety of using e-cigarettes [23]. Meanwhile, most e-cigarette manufacturers and retailers have been actively using social media as a platform to promote products and collect feedback from consumers. The role of social media in marketing is strengthened by limitations on advertising and marketing of tobacco products [23].

Research has demonstrated that such social media and Internet data could be used to evaluate the diffusion of health products and health behaviors related to e-cigarettes [22-25]. A cross-sectional study revealed that e-cigarette–related Twitter posts were overwhelmingly commercial, with frequent mentions of smoking cessation [23]. Another study on the retweet network of e-cigarette–related posts validated the use of social media as a proxy filter for marketing messages [26]. Another study using YouTube data categorized e-cigarette–related videos by attitudes and types, and showed that most videos held positive views of e-cigarettes [25]. A content analysis of Reddit posts demonstrated that flavor-related social media information could reflect smokers’ interest in e-cigarette products containing these flavors [22]. Several empirical studies examining flavor-related e-cigarette marketing on social media found that posts that mentioned flavor received more positive comments and had a higher chance to be reposted than those without flavors [13,26,27]. However, previous studies did not recognize the possibility that the influence on information propagation may vary across different flavors. An in-depth understanding of the information propagation of posts mentioning specific flavors could inform practical marketing strategies for retailers and provide policy suggestions for regulatory authorities. Our research aimed to address this challenge to characterize the influence of flavors on the information propagation of e-cigarette–related posts on social media.

## Methods

### Data Description

In this study, we collected a comprehensive dataset from Facebook (Facebook, Inc), the biggest social media platform. In addition to social networking functions, Facebook allows individuals or organizations to create (public) Pages for users to form communities for various purposes. Facebook Pages have been widely used by companies (including all major e-cigarette manufacturers and retailers) as a platform for marketing and maintaining customer relations [28]. Those Pages also represent active communities for e-cigarette users to discuss topics related to e-cigarettes, including flavors, promotional campaigns, the pros and cons of consuming e-cigarettes, and safety issues. The rich discussions about e-cigarettes in public Pages provide an ideal data source to identify consumers’ perceptions and preferences. and the diffusion of multiple flavors.

Based on keywords generated by domain experts (as Textbox 1 shows), we retrieved a set of e-cigarette–related Facebook Pages through Facebook’s application programming interface (API). We derived the keywords in Textbox 1 from the combination of domain expertise and the published literature [29-31]. For consistency, we manually extracted the Pages related to smoking promotion run by e-cigarette manufacturers and retailers. Finally, we collected the full information of all posts with comments. In total, we collected 7132 e-cigarette–related Facebook Pages with 765,321 posts up to April 24, 2015. Of these posts, 86.68% (663,357/765,321) were generated during 2013 to 2015. A post may receive comments and likes from Facebook users and can be shared by users (to their own Facebook timelines). We collected 2,737,840 comment records and 17,671,614 like records. For each post, we collected the Page identifier (ID), post ID, user ID (who posted the post), time when the post was created, textual content, and the records of comments, likes, and shares. For each comment record, we collected user ID (who posted the comment), time when it was created, and textual content. For each like record, we collected user ID (who clicked the Like button of the original post) and time of clicking the Like button. In total, we identified 1,414,240 unique user IDs. Then, we collected the full public profiles of these users, including their screenname, language, location, and sex. To be consistent, we chose 384,792 posts generated by users with the label “en_US,” indicating they were English-speaking Facebook users located in the United States.

**E-cigarette–related keywords for data collection.**

electronic cigarette, disposable cigarette, e-cig, e-cigarette, rechargeable cigarette, rechargeable kits, flavor cartridge, vaporizer, vaporized, vapor, vaping, mod, apv, refill cartridges, vaping pen, refills, cigalikes, mechs, vape pen, electronic pipe, cartomizer, clearomizer, atomizer, hookah, electronic hookah, shisha, electronic shisha, e-hookah, e-shisha, electronic cigar, e-cigar, electronic juice, electronic liquid, e-juice, e-liquid, electronic joint, e-joint, electronic spliff, e-spliff, vape, vaping, istick, coil tank, coil, rda

### Variables Description

We characterized the influence of different flavors on the information propagation patterns using regression models. In this section, we explain the variables for candidate regression models.

**Figure 1:**
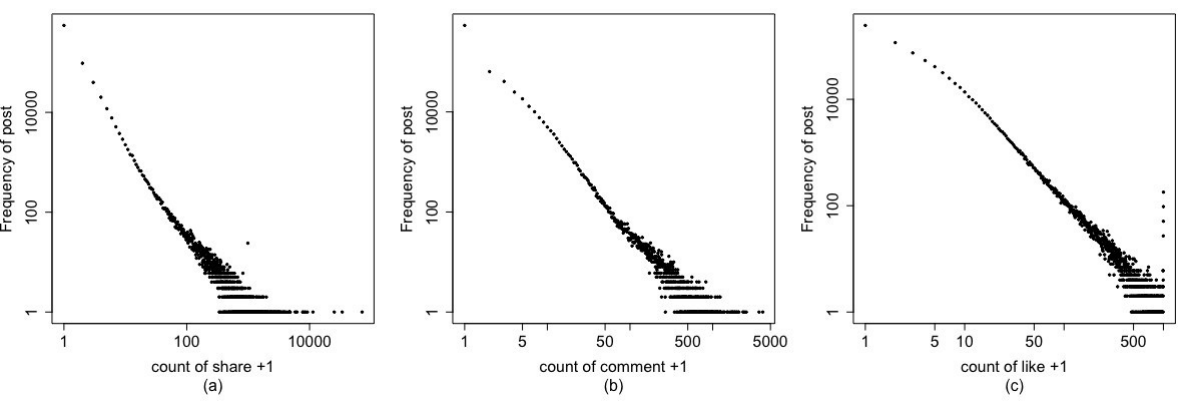
Distributions of (a) shares, (b) comments, and (c) likes. We added 1 to each value on the x-axis to avoid the logarithm of zeros on the horizontal axis.

When users browse posts, photos, and other information on Facebook, they can click the Like button for that information, post comments, and share the information to their own timelines. The frequency of a post being shared and liked, and the number of comments received, are explicit proxy measures of information propagation. Figure 1 shows the distributions of these 3 variables (note that we added 1 to each value on the x-axis to avoid the logarithm of zeros on the horizontal axis). In general, we observed a power law–shaped curve in these distributions. This “rich-get-richer” effect indicated that the popularity of a post and the information propagation were unevenly distributed, with most of the posts being seldom shared, commented on, or liked, while a small number of popular posts received a huge number of shares, comments, and likes.

When Facebook user *A* shares a post published on a Facebook Page, this post then appears in *A* ’s timeline, as well as on the newsfeed (home page) of *A* ’s friends. Therefore, the sharing behavior presents the information propagation from the Page to the user and the user’s friends. If one of *A* ’s friends, *B*, also shared the same post after reading it from the newsfeed (because *A* shared it), our data collection also captured this new sharing behavior. It is impossible to differentiate the original shares and subsequent shares caused by specific propagation paths through the newsfeed, because Facebook’s API prohibits the collection of friendship information. On the other hand, 2 additional proxies of information propagation, comments and likes generated by user *A*, will not be explicitly presented to *A* ’s friends. Therefore, data on post-sharing behaviors is the most effective and reliable proxy to identify, track, and model information propagation on Facebook [26,32]. In this study, we calculated the frequency of being shared by Facebook users for each post (denoted as Shares) as the dependent variable (representing information propagation) in regression models.

**Figure 2:**
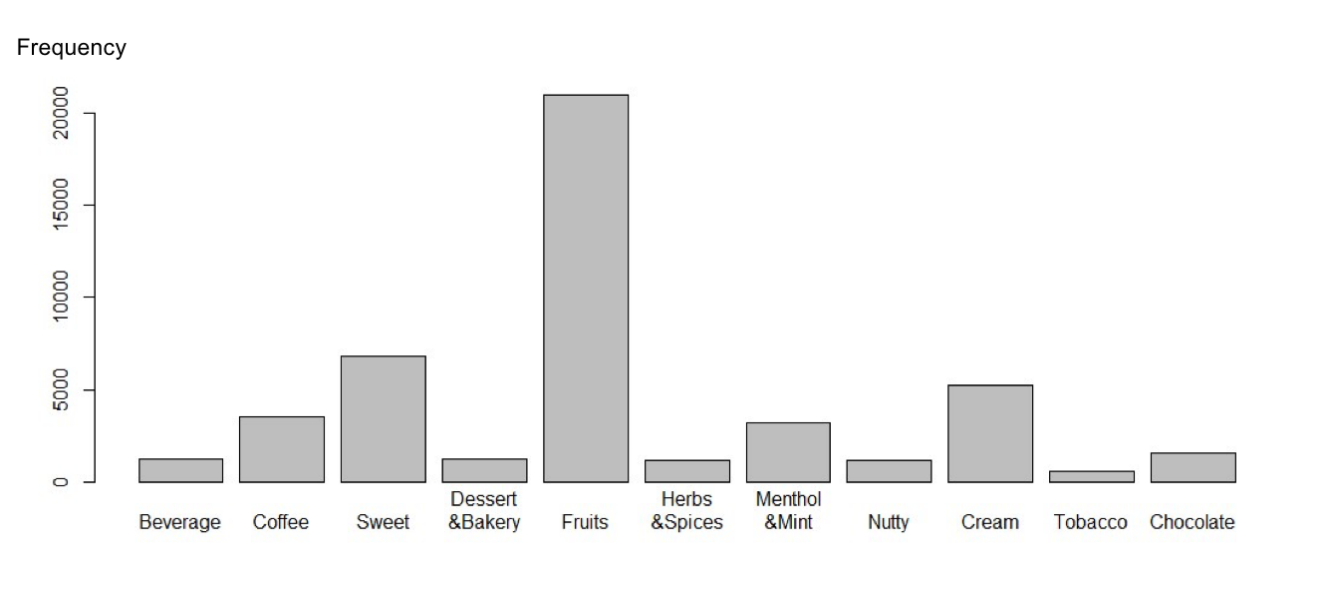
Occurrences of the 11 flavor categories in e-cigarette–related Facebook posts.

Because of the lack of regulations, manufacturers and retailers do not have a universal flavor classification system. Researchers have used questionnaires and data mining methods to identify a set of the main categories of e-cigarette flavors [12,22,27]. Borrowing and evaluating these categorizations, we identified 11 categories of flavors of e-cigarettes in our dataset: beverage, coffee, sweet, dessert & bakery, fruits, herbs & spices, menthol & mint, nutty, cream, tobacco, and chocolate. We also identified a set of keywords for each category (eg, coke and pepsi are keywords for beverage). It is worth mentioning that the content of a post could contain more than one flavor. Figure 2 shows the distribution of posts that mentioned flavors. Among all of the flavors, fruits was the most popular, followed by sweet and cream.

To characterize the influence of these 11 categories of flavors on information propagation (measured by the frequency of being shared), we introduced 11 binary variables for flavor categories. Each binary variable represented the existence of keywords belonging to the corresponding flavor category.

To avoid bias, we introduced a set of nonflavor-related variables that could have influenced information propagation and correlated with flavor-related variables. In Facebook Pages, manufacturers and retailers often promote their products by offering consumers rewards and gifts by lottery among those users who liked, shared, or commented on the posts. Obviously, such promotional activities would largely increase the appeal of posts to the users. We first identified promotion-related posts based on a set of keywords related to promotions (eg, reward, share, gifts, and free). Then, we added the binary dummy variable promotion to represent the existence of promotion in the corresponding post.

The activeness of a Facebook Page is often associated with its popularity. In general, the more active a Facebook Page is, the more frequently its posts can be viewed by users. To capture this effect, we used the count of posts in a Facebook Page as an independent variable, Posts, to measure the activeness of the Page. In addition, the level of user engagement is diverse because of many unknown factors (eg, the popularity of the brand). To differentiate the influence of flavors and the Page-specific user engagement level, we calculated the average number of shares per post of each Page as a control variable, average share.

The topics conveyed by posts could have a significant influence on information propagation. To capture the potential effect of topics, we employed the commonly used latent Dirichlet allocation, an unsupervised learning model for topic modeling, to extract 3 topics hidden in the text of posts: details about products (product), methods of consuming e-cigarettes (method), and other related discussions (other). Table 1 lists the top 10 most frequent words for each topic. For more details about topic modeling, please refer to Multimedia Appendix 1.

The content of posts often contained URLs and hashtags. URLs provide external information related to the posts. Hashtags are used to help Facebook users label and identify posts with specific topics. Both URLs and hashtags have been found to be associated with the likelihood of information propagation [33]. We introduced 2 control variables, URL mention and Hashtag, to represent the existence of URLs and hashtag labels, respectively. Table 2 summarizes all variables, and Multimedia Appendix 1 summarizes Pearson correlation coefficients.

**Table 1:** Top 10 most frequent words for each topic.

Rank	Topic 1: product (details about e-cigarettes)		Topic 2: method (methods of e-cigarette consumption)		Topic 3: others (related discussions)	
	Words	Frequency (x 10^–2^)	Words	Frequency (x 10^–2^)	Words	Frequency
1	new	1.76	vape	1.80	tobacco	.77
2	now	1.26	get	1.69	Smoking	.73
3	flavor	.88	vapor	1.22	know	.06
4	stock	.84	free	1.17	smoke	.53
5	mod	.83	hookah	.89	thank	.52
6	available	.77	juice	.85	like	.52
7	flavors	.66	like	.79	want	.50
8	2 (oz.)	.61	vaping	.79	time	.50
9	1 (oz.)	.58	everyone	.78	help	.40
10	battery	.48	happy	.75	vaping	.40

**Table 2:** Summary statistics of dependent and independent variables and control variables.

Variables			Mean (SD)		Minimum	Maximum
**Dependent variable**						
	Shares		7.10 (154.85)		0	71,668
**Independent variables, mean (SD) x 10^–2^**						
	Beverage		0.33 (5.72)		0	1
	Coffee		0.91 (9.59)		0	1
	Sweet		1.77 (13.20)		0	1
	Dessert & bakery		0.33 (5.73)		0	1
	Fruits		5.44 (22.68)		0	1
	Herbs & spices		0.30 (5.51)		0	1
	Menthol & mint		0.84 (9.12)		0	1
	Nutty		0.31 (5.58)		0	1
	Cream		1.37 (11.64)		0	1
	Tobacco		0.16 (4.04)		0	1
	Chocolate		0.41 (6.39)		0	1
**Control variables**						
	Promotion		0.04 (0.19)		0	1
	Posts		618.11 (780.06)		1	5,980
	Average share		7.10 (18.51)		0	2,258
	Topic 1		0.33 (0.08)		0.03	0.97
	Topic 2		0.34 (0.07)		0.01	0.91
	Topic 3		0.33 (0.07)		0.01	0.94
	URL mention		0.14 (0.35)		0	1
	Hashtag		0.12 (0.33)		0	1

### Model Selection

We evaluated a set of regression models for count data to characterize the influence of flavors on e-cigarette–related information propagation. We used Stata software version 12.0 (StataCorp LLC) to estimate parameters.

The Poisson regression model is the most common method to model count data. It assumes that the mean and variance of the dependent variable are equal; thus, we needed to test the overdispersion effect of the data to confirm the assumption. We used the following *z* score test to evaluate whether the overdispersion effect in the Poisson regression model was significant enough to violate the fundamental assumption [34]: *z*=[(*y* –µ)^2^– *y*]/µ√2, where y is the dependent variable and µ is the expectation. We obtained a *z* score of 1228.886 with a *t* probability of .04. This indicated that there was a significant overdispersion effect and the Poisson regression model was not ideal for these data. This was also reflected by the poor goodness-of-fit, as indicated by the large value of the Akaike information criterion (AIC).

The negative binomial regression model is widely used to resolve the overdispersion problem by relaxing the Poisson assumption through adding constant dispersion parameter α. However, the negative binomial assumption is difficult to meet when excessive zeros exist in dependent variables.

In our study, we found that 61.1% of observations of the dependent variables were zero. To handle excessive zeros, we used the zero-inflated regression model and the hurdle regression model. In the zero-inflated model, the dependent variable is modeled as a mixture of the count data model (eg, Poisson regression model, negative binomial regression model) and a separate Bernoulli distribution. In the hurdle model, there are 2 components to model the dependent variable: positives are generated by a truncated-at-zero count data model, and zeros are generated by a Bernoulli distribution. Both models can overcome the limit of standard count data models, which assume that zeros and positives are both generated by the same process.

## Results

We evaluated the performance of the proposed models using our data (as Table 3 shows with coefficients and *P* values). We observed that the negative binomial regression, the hurdle negative binomial regression model, and the zero-inflated negative binomial regression model performed significantly better than the Poisson model. The hurdle negative binomial regression model had the best performance as indicated by the lowest AIC/n. Therefore, we selected the hurdle negative binomial regression model as the base model to characterize the relationship between the independent and dependent variables. Then, we examined the influence of nonflavor-related variables on the fit of flavor-related variables; Table 4 presents the final model, with coefficients and *P* values.

Regarding the results of the base and final regression models presented in Table 4, the first set of columns show our estimates of a specification of the initial model with only flavors as the independent variables. The negative and significant coefficients for coffee, fruits, and tobacco suggested that the existence of these flavors tended to reduce the chance of propagation of the corresponding e-cigarette–related information.

The additional control variables in the second and third sets of columns modified the estimates of flavors’ influence on information propagation. Specifically, the estimates of tobacco became nonsignificant, indicating that its effect was weakened after adding control variables. The significance of herbs & spices, dessert & bakery and cream became visible with the addition of promotion. Most control variables were significant. Particularly, the large *z* score and coefficient of promotion suggested that promotion was the dominating variable among all the independent variables. This is reasonable because the promotions in a post would greatly increase its chance of being shared by users.

**Table 3:** Results of regression models.

Variables		Model							
		Poisson		Negative binomial		Zero-inflated negative binomial		Hurdle negative binomial	
		Coefficient^a^	*P* value	Coefficient^a^	*P* value	Coefficient^a^	*P* value	Coefficient^a^	*P* value
**Independent variables**									
	Beverage	–0.65	.01	–0.11	.29	0.66	.02	–0.23	.12
	Coffee	–0.85	<.001	–0.02	.73	0.14	.34	–0.15	.21
	Sweet	–0.11	.34	–0.19	.006	–0.60	<.001	–0.34	.001
	Dessert & bakery	–0.49	.003	–0.28	<.001	–0.70	<.001	–0.52	<.001
	Fruits	–0.29	<.001	–0.16	<.001	–0.13	.008	–0.24	<.001
	Herbs & spices	–0.07	.87	–0.57	<.001	–0.64	<.001	–0.60	<.001
	Menthol & mint	–0.35		–0.20	.003	–0.12	.21	–0.08	.46
	Nutty	0.11	.71	0.15	.17	–0.27	.11	0.002	.99
	Cream	0.09	.49	–0.13	.13	–0.79	<.001	–0.15	.27
	Tobacco	–1.22	<.001	–0.44	<.001	0.33	.14	–0.55	.001
	Chocolate	–0.09	.59	0.03	.82	–0.36	.11	0.09	.65
**Control variables**									
	Promotion	3.12	<.001	3.06	<.001	2.01	<.001	3.16	<.001
	Posts (Coefficient x 10^–4^)	3.46	<.001	2.78	<.001	1.18	<.001	3.58	<.001
	Average share (Coefficient x 10^–2^)	.48	<.001	6.67	<.001	4.18	<.001	10.17	<.001
	Topic 1	6.15	<.001	1.61	<.001	0.79	<.001	1.39	<.001
	Topic 2	7.75	<.001	1.11	<.001	1.42	<.001	1.17	<.001
	URL mention	0.09	.12	0.25	<.001	0.27	<.001	0.19	<.001
	Hashtag	–0.39	<.001	–0.05	.25	0.01	.75	–0.02	.72
Intercept		–3.73	<.001	–0.97	<.001	–0.53	<.001	–18.79	<.001
Model zero		No		No		Yes		Yes	
Model dispersion		No		Yes		Yes		Yes	
AIC/n^b^		33.74		3.20		3.24		3.14	

^a^Estimate of coefficient for each variable in the model.

^b^AIC: Akaike information criterion. The hurdle negative binomial regression model had the best performance as indicated by the lowest AIC/n (AIC value divided by number of observation).

**Table 4:** Results of the hurdle negative binomial regression models.

Variables		Model 1^a^ (n=384,792^b^)		Model 2^c^ (n=384,792^b^)		Model 3^d^ (n=384,792^b^)		Without promotion (n=370,670^b^)		With promotion (n=14,122^b^)	
		Coefficient	*P* value	Coefficient	*P* value	Coefficient	*P* value	Coefficient	*P* value	Coefficient	*P* value
**Independent variables**											
	Beverage	0.05	.94	0.64	.36	–0.23	.12	–0.20	.18	–1.38	<.001
	Coffee	–1.69	<.001	–0.69	.003	–0.15	.21	–0.10	.40	–1.53	<.001
	Sweet	0.11	.496	–0.31	.06	–0.34	.001	–0.35	.002	–0.04	.81
	Dessert & bakery	–0.46	.09	–0.64	.04	–0.52	<.001	–0.57	<.001	–0.08	.78
	Fruits	–0.31	.004	–0.67	<.001	–0.24	<.001	–0.25	<.001	–0.07	.48
	Herbs & spices	0.20	.70	–1.19	<.001	–0.60	<.001	–0.61	<.001	0.14	.69
	Menthol & mint	–0.24	.34	–0.01	.97	–0.08	.46	–0.06	.58	–0.24	.55
	Nutty	–0.06	.86	0.01	.97	0.002	.99	–0.04	.79	0.65	.08
	Cream	0.16	.38	–0.39	.02	–0.15	.27	–0.19	.18	0.52	.007
	Tobacco	–1.65	<.001	–0.94	.06	–0.55	.001	–0.54	.001	–0.66	.10
	Chocolate	–0.14	.63	0.28	.57	0.09	.65	0.10	.62	0.13	.59
**Control variables**											
	Promotion			3.42	<.001	3.16	<.001				
	Posts (Coefficient x 10^–4^)			—^e^	—	3.58	<.001	3.59	<.001	2.10	<.001
	Average share (Coefficient x 10^–2^)			—	—	10.17	<.001	.11	<.001	3.20	<.001
	Topic 1			—	—	1.39	<.001	1.19	<.001	3.54	<.001
	Topic 2			—	—	1.17	<.001	0.73	.008	10.26	<.001
	URL mention			—	—	0.19	<.001	0.20	<.001	0.01	.89
	Hashtag			—	—	–0.02	.72	0.005	.94	–0.12	.24
Intercept		–18.18	<.001	–16.96	<.001	–18.79	<.001	–15.38	<.001	–2.42	<.001

^a^Estimates of a specification of the initial model with only flavors as the independent variables.

^b^Number of observations is defined by n.

^c^Estimates of flavors’ influence on information propagation modified by adding promotion only.

^d^Estimates of flavors’ influence on information propagation modified by additional control variables.

^e^Variable not used in the second "All data" model.

Although promotion had the major predictive power to explain e-cigarette–related information propagation, it caused an adverse effect on other, less-powerful independent variables. The effect of promotion was too overwhelming, making the observed influence of flavors unreliable. Therefore, we split the observations into 2 parts, 370,670 posts without promotions and 14,122 posts with promotions, and fit the model separately to eliminate the dominant effect. The fourth and fifth sets of columns of Table 4 show the results. We found that the results in the fourth column (without promotion effects) were similar to those in the third column (base model), but not the fifth column. The difference between the fourth and fifth columns suggested that these two types of posts (with and without promotions) had different sharing patterns, making the base model inappropriate. Eventually, we chose the fourth column (without promotion) to be the final model.

In the final model, sweet, dessert & bakery, fruits, herbs & spices, and tobacco had a significant negative influence on the propagation of e-cigarette–related posts. The chance of a post being shared was lower when the post contained keywords belonging to these 5 flavor categories, indicating the lack of users’ interests in these flavors. This is contradictory to previous research. Although previous studies were different from our research in that they did not categorize flavors, our finding still implies the general lack of interest in all flavors of e-cigarettes on Facebook, because we did not find a positive significance of any flavor category.

A closer look at these posts helped us identify a possible cause of the low interest in flavors: users may have had concerns about the safety issues of the flavoring additives in e-cigarettes. In the United States, flavoring additives approved by the US Food and Drug Administration were only tested for consumption in food and beverages. The safety of consuming these flavoring additives through inhalation (as with e-cigarettes) is not well tested or regulated [21]. In addition, certain flavors may contain untested elements that harm human health. For example, studies showed that many e-cigarette flavorings contained an excessive amount of aldehyde, which is the primary irritant of the mucosal tissue in the respiratory tract [21]. The negative significance of the herbs & spices flavor in our regression model echoes recent studies showing the cytotoxicity of chemicals used in this type of flavor [15,16]. Similarly, the negative significance of sweet-related flavors (eg, sweet, dessert & bakery, and fruits) also echoes another study indicating the association between the use of certain chemicals in sweet-flavored e-cigarettes and respiratory diseases [17]. These potential risks associated with flavors could be a possible reason for the lower popularity of e-cigarette flavors among Facebook users.

## Discussion

### Principal Findings

This study was, to our knowledge, the first data-driven research to characterize the influence of categorized flavors in e-cigarette–related information propagation on social media. Surprisingly, we found that flavors did not enhance the popularity of e-cigarette–related information. Certain flavors even reduced the popularity, indicating users’ lack of interest in flavors and potential concern about the safety issues of flavoring additives. For manufacturers and retailers, this study suggests that promoting e-cigarettes with flavors is not an effective marketing approach. For regulatory authorities and policy makers, this study suggests that new policies with updated regulations and restrictions on flavors are needed, for the sake of the health of e-cigarette users.

### Limitations

Our study had several limitations. First, data derived from social media are obviously biased. We only studied English-language content posted by US users. Higher-resolution data with detailed demographic information could improve the practical value of this research significantly. In addition, for consistency, Facebook Pages in our dataset were mainly from parties making or selling e-cigarettes, with a commercial focus. The information propagation patterns on Facebook Pages of nongovernmental organizations and health authorities could be different, thus needing further studies.

Second, information propagation is only one aspect of examining the diffusion of health products. Analyzing the content of information could help us extract users’ opinions and emotions while discussing e-cigarette–related topics. Content analysis could also help us understand the root cause of the lack of interest in flavors revealed in this study.

Third, we evaluated the propagation of information by counting the number of shares of each post. This method measures the scale of propagations well, but could not measure the depth of propagations accurately. The Facebook API prohibited us from retrieving more detailed information about the accurate propagation path because of privacy concerns. There is a need for future research on the depth of information propagation using other data sources (eg, Twitter and Reddit).

Fourth, more data-driven medical research is critically needed to identify the root cause of the lower popularity of certain flavors of e-cigarettes.

### Conclusions

This study found that mentions of flavors in posts did not enhance the popularity of e-cigarette–related information. There are several future works that we will pursue. First, we plan to validate the findings of this study using the data of other social media platforms under different cultural and language settings. In addition, we will develop state-of-the-art text mining methodologies to identify social media users’ opinions of flavors and the use of e-cigarettes with different flavors. We will also develop probabilistic topic models to identify various topics related to e-cigarettes for smoking surveillance. This line of social media research has great potential to help e-cigarette manufacturers, retailers, regulatory authorities, and policy makers understand the behaviors and opinions of e-cigarette users. This study demonstrated the potential of using social media data to understand the behaviors of e-cigarette users through an empirical study of flavors, and it calls for more research from other perspectives to fulfill the potential of this valuable big data source.

## Appendix

Multimedia Appendix 1 Supplementary tables.

## Abbreviations

AIC: Akaike information criterion
API: application programming interface
ID: identifier
