# Supplementary material for: Influence of Flavors on the Propagation of E-Cigarette–Related Information: Social Media Study
Source: JMIR Public Health Surveill. 2018 Mar 23;4(1):e27. doi: 10.2196/publichealth.7998 (PMC5889495; doi:10.2196/publichealth.7998)
Supplement: Multimedia Appendix 1 [file publichealth_v4i1e27_app1.pdf]

Influence of Flavors on the Propagation of Electronic Cigarette Related Information: A Social Media Study

Supplementary materials

Jiaqi Zhou, MSc, Department of Systems Engineering and Engineering Management, City University of Hong Kong, Kowloon, Hong Kong SAR, China. Shenzhen Research Institute of City University of Hong Kong, Shenzhen, Guangdong 518057, China.

Qingpeng Zhang, PhD (corresponding author), Department of Systems Engineering and Engineering Management, City University of Hong Kong, Kowloon, Hong Kong SAR, China. Shenzhen Research Institute of City University of Hong Kong, Shenzhen, Guangdong 518057, China.

Daniel Dajun Zeng, PhD, Department of Management Information Systems, The University of Arizona, Tucson, AZ 85721, USA. Institute of Automation, Chinese Academy of Sciences, Beijing 100190, China.

Kwok-Leung Tsui, PhD, Department of Systems Engineering and Engineering Management, City University of Hong Kong, Kowloon, Hong Kong SAR, China.

Corresponding author: Qingpeng Zhang, PhD (Email: qingpeng.zhang@cityu.edu.hk; Phone: +852-3442-4727; Fax: +852-3442-0173), Department of Systems Engineering and Engineering Management, City University of Hong Kong, Kowloon, Hong Kong SAR, China. Shenzhen Research Institute of City University of Hong Kong, Shenzhen, Guangdong 518057, China.

Supplementary Table 1. The detailed fitting results of the hurdle negative binomial regression model

(column 4 in Table 5).

		Coef.	Std. Err.	z	P>|z|	95% Conf.Interval	
Independent variables	Beverage	-0.19712	0.147505	-1.34	0.181	-0.48623	0.091984
	Coffee	-0.10124	0.119263	-0.85	0.396	-0.33499	0.132509
	Sweet	-0.34874	0.113057	-3.08	0.002	-0.57033	-0.12715
	Dessert&Bakery	-0.57427	0.126166	-4.55	0	-0.82155	-0.32699
	Fruits	-0.25275	0.069912	-3.62	0	-0.38977	-0.11572
	Herb&Spices	-0.60725	0.11795	-5.15	0	-0.83843	-0.37608
	Menthol&Mint	-0.06217	0.113549	-0.55	0.584	-0.28472	0.160386
	Nutty	-0.04144	0.157661	-0.26	0.793	-0.35045	0.267568
	Cream	-0.19296	0.145286	-1.33	0.184	-0.47771	0.091798
	Tobacco	-0.53807	0.163306	-3.29	0.001	-0.85814	-0.218
	Chocolate	0.104311	0.209455	0.5	0.618	-0.30621	0.514836
Control variables	Promotion #Post	-0.000359	.0000229	15.66	0	0.000314	0.000404
	Avg_share	0.105603	0.002722	38.79	0	0.100267	0.110939
	Topic 1	1.188897	0.238384	4.99	0	0.721672	1.656122
	Topic 2	0.734471	0.277418	2.65	0.008	0.190740	1.278202
	URLmention	0.200702	0.041861	4.79	0	0.118656	0.282748
	Hashtag	0.004997	0.0638	0.08	0.938	-0.12005	0.130043
	Intercept	-15.3811	0.159009	-96.73	0	-15.6927	-15.0694

Supplementary Table 1 is the detailed fitting results of the chosen final model (column 4 in Table 5). We split the observations and used those posts without promotions to fit the hurdle negative binomial regression model.

Supplementary Table 2. The top ten most frequent words for two topics.

Topic 1		Topic 2	
words	frequency	words	frequency
vape	0.014303	like	0.008333
new	0.011112	get	0.008052
now	0.009923	free	0.006169
vapor	0.008759	great	0.006086
hookah	0.006391	flavor	0.005405
check	0.006183	new	0.005173
stock	0.005883	juice	0.005126
mod	0.005808	see	0.005084
2 (oz.)	0.005777	time	0.00506
1 (oz.)	0.005678	tobacco	0.004965

Top ten most frequent words for each topics extracted by LDA model. There are two topics in this table.

Supplementary Table 3. Hurdle negative binomial regression model with two topics.

		Coef.	Std. Err.	z	P>|z|	95% Conf.Interval	
Independent variables	Beverage	-0.15883	0.148543	-1.07	0.285	-0.44997	0.13231
	Coffee	-0.07258	0.12317	-0.59	0.556	-0.31399	0.168831
	Sweet	-0.29027	0.114275	-2.54	0.011	-0.51424	-0.06629
	Dessert&Bakery	-0.5208	0.128841	-4.04	0	-0.77332	-0.26828
	Fruits	-0.20112	0.071928	-2.8	0.005	-0.3421	-0.06015
	Herb&Spices	-0.58638	0.120219	-4.88	0	-0.82201	-0.35076
	Menthol&Mint	-0.02575	0.115573	-0.22	0.824	-0.25227	0.200764
	Nutty	-0.00281	0.158229	-0.02	0.986	-0.31294	0.30731
	Cream	-0.12702	0.135039	-0.94	0.347	-0.3917	0.137649
	Tobacco	-0.52285	0.160359	-3.26	0.001	-0.83715	-0.20856
	Chocolate	0.180539	0.220514	0.82	0.413	-0.25166	0.612739
Control variables	Promotion	-					
	#Post	0.000351	2.28E-05	15.39	0	0.000306	0.000396
	Avg_share	0.106205	0.002667	39.82	0	0.100978	0.111433
	Topic 1	0.852842	0.186109	4.58	0	0.488075	1.217608
	URLmention	0.202839	0.042468	4.78	0	0.119604	0.286074
	Hashtag	0.003194	0.065305	0.05	0.961	-0.1248	0.13119
	Intercept	-21.3906	0.100206	-213.47	0	-21.587	-21.1942

Supplementary Table 3 is the fitting result of hurdle negative binomial regression model with two topics. Because of the collinearity, we deleted topic 2 while fitting the model.

Supplementary Table 4. The top ten most frequent words for three topics.

Details about e-cig<br>Topic 1		Methods of taking e-cig<br>Topic 2		Others<br>Topic 3	
words	frequency	words	frequency	words	frequency
new	0.017568	**vape**	0.018026	**tobacco**	0.007689
now	0.012615	get	0.016905	**Smoking**	0.007344
**flavor**	0.008788	**vapor**	0.012228	know	0.006021
stock	0.008433	free	0.011691	**smoke**	0.005326
**mod**	0.008325	**hookah**	0.008930	thank	0.005219
available	0.007714	juice	0.008486	like	0.005212
**flavors**	0.006610	like	0.007885	want	0.005037
**2 (oz.)**	0.006100	**vaping**	0.007868	time	0.004976
**1 (oz.)**	0.005840	everyone	0.007776	help	0.004044
**battery**	0.004830	happy	0.007503	vaping	0.004029

Top ten most frequent words for each topics extracted by LDA model. There are three topics in this table. According to the high frequency words, we find that topic 1 talks about the details about e-cigarette such as the flavor, mod and the dose (the number 1 and 2 mean the dose of e-liquid), topic 2 is about hookah vape, and topic 3 is the general talking of e-cigarettes. Because of the collinearity of these three topics, we delete topic 3 in the regression model.

Supplementary Table 5. Hurdle negative binomial regression model with three topics.

		Coef.	Std. Err.	z	P>|z|	95% Conf.Interval	
Independent variables	Beverage	-0.19712	0.147505	-1.34	0.181	-0.48623	0.091984
	Coffee	-0.10124	0.119263	-0.85	0.396	-0.33499	0.132509
	Sweet	-0.34874	0.113057	-3.08	0.002	-0.57033	-0.12715
	Dessert&Bakery	-0.57427	0.126166	-4.55	0	-0.82155	-0.32699
	Fruits	-0.25275	0.069912	-3.62	0	-0.38977	-0.11572
	Herb&Spices	-0.60725	0.11795	-5.15	0	-0.83843	-0.37608
	Menthol&Mint	-0.06217	0.113549	-0.55	0.584	-0.28472	0.160386
	Nutty	-0.04144	0.157661	-0.26	0.793	-0.35045	0.267568
	Cream	-0.19296	0.145286	-1.33	0.184	-0.47771	0.091798
	Tobacco	-0.53807	0.163306	-3.29	0.001	-0.85814	-0.218
	Chocolate	0.104311	0.209455	0.5	0.618	-0.30621	0.514836
Control variables	Promotion #Post	-0.000359	.0000229	15.66	0	0.000314	0.000404
	Avg_share	0.105603	0.002722	38.79	0	0.100267	0.110939
	Topic 1	1.188897	0.238384	4.99	0	0.721672	1.656122
	Topic 2	0.734471	0.277418	2.65	0.008	0.190740	1.278202
	URLmention	0.200702	0.041861	4.79	0	0.118656	0.282748
	Hashtag	0.004997	0.0638	0.08	0.938	-0.12005	0.130043
	Intercept	-15.3811	0.159009	-96.73	0	-15.6927	-15.0694

Supplementary Table 5 is the fitting result of hurdle negative binomial regression model with three topics. Because of the collinearity, we deleted topic 3 while fitting the model.

Supplementary Table 6. The top ten most frequent words for four topics.

Topic 1		Topic 2		Topic 3		Topic4	
words	frequency	words	frequency	words	frequency	words	frequency
now	0.018609784	smoking	0.009074	new	0.018113	vape	0.016018
new	0.014812965	tobacco	0.008636	juice	0.014587	free	0.009979
stock	0.011138696	thank	0.007615	flavor	0.01213	hookah	0.009564
mod	0.010995441	know	0.007441	flavors	0.010963	everyone	0.00909
get	0.010746983	time	0.007228	vape	0.010393	happy	0.008568
2 (oz.)	0.008654116	smoke	0.006581	great	0.007615	vapor	0.008458
check	0.008448747	want	0.006224	bottle	0.007588	vaping	0.00831
1 (oz.)	0.008400063	like	0.005453	try	0.007277	open	0.007819
available	0.008325637	help	0.004998	week	0.006181	share	0.0076
battery	0.006379383	vaping	0.004886	soon	0.006002	post	0.00677

Top ten most frequent words for each topics extracted by LDA model. There are four topics in this table.

Supplementary Table 7. Hurdle negative binomial regression model with four topics.

		Coef.	Std. Err.	z	P>|z|	95% Conf.Interval	
Independent variables	Beverage	-0.13622	0.14617	-0.93	0.351	-0.42271	0.150266
	Coffee	-0.11525	0.124253	-0.93	0.354	-0.35878	0.128282
	Sweet	-0.31252	0.107644	-2.9	0.004	-0.52349	-0.10154
	Dessert&Bakery	-0.5358	0.127414	-4.21	0	-0.78552	-0.28607
	Fruits	-0.17293	0.057805	-2.99	0.003	-0.28622	-0.05963
	Herb&Spices	-0.44566	0.118382	-3.76	0	-0.67768	-0.21363
	Menthol&Mint	0.107014	0.11042	0.97	0.332	-0.10941	0.323434
	Nutty	0.006791	0.154758	0.04	0.965	-0.29653	0.31011
	Cream	-0.04696	0.133839	-0.35	0.726	-0.30927	0.215364
	Tobacco	-0.39438	0.184801	-2.13	0.033	-0.75659	-0.03218
	Chocolate	0.240355	0.233986	1.03	0.304	-0.21825	0.698958
Control variables	Promotion #Post	-0.000371	1.96E-05	18.91	0	0.000333	0.00041
	Avg_share	0.096826	0.002483	39	0	0.09196	0.101692
	Topic 1	-3.72437	0.222349	-16.75	0	-4.16017	-3.28858
	Topic 2	-3.77828	0.217237	-17.39	0	-4.20406	-3.35251
	Topic 3	-3.05164	0.31342	-9.74	0	-3.66593	-2.43735
	URLmention	0.242702	0.03678	6.6	0	0.170615	0.314788
	Hashtag	-0.11745	0.055095	-2.13	0.033	-0.22543	-0.00947
	Intercept	-18.174	0.158867	-114.4	0	-18.4854	-17.8627

Supplementary Table 7 is the fitting result of hurdle negative binomial regression model with four topics. Because of the collinearity, we deleted topic 4 while fitting the model.

Supplementary Table 8. Hurdle negative binomial regression model with one consolidated flavor variable.

		Coef.	Std. Err.	z	P>|z|	[95% Conf. Interval]	
Independent variable	Flavor	-0.48906	0.066618	-7.34	0	-0.61963	-0.35849
Control variables	Promotion	-					
	#Post	0.000364	2.31E-05	15.75	0	0.000319	0.000409
	Avg_share	0.105519	0.00273	38.65	0	0.100169	0.11087
	Topic 1	1.112665	0.237093	4.69	0	0.64797	1.577359
	Topic 2	0.752316	0.278586	2.7	0.007	0.206297	1.298334
	URLmention	0.200275	0.041881	4.78	0	0.11819	0.282361
	Hashtag	0.003873	0.064332	0.06	0.952	-0.12222	0.129961
	Intercept	-18.3189	0.16531	-110.82	0	-18.6429	-17.9949

Supplementary Table 8 lists the fitting result of the hurdle negative binomial regression model while consolidating all flavors into one variable.

Supplementary Table 9. Pearson correlation coefficient between covariates

	Beverages	Coffee	Sweet	Dessert&Bakery	Fruits	Herbs&Spices	Menthol&Mint	Nutty	Cream	Tobacco	Chocolate	Promotion	#Post	Avg_share	Topic 1	Topic 2	URLmention	Hashtag
Beverages	1	0.09	0.07	0.03	0.11	0.01	0.11	0.04	0.09	0.01	0.1	0	-0.01	0	0.05	-0.02	-0.01	0
Coffee	0.09	1	0.07	0.05	0.1	0.02	0.1	0.06	0.09	0.02	0.12	-0.01	-0.01	-0.02	0.05	-0.02	0	0
Sweet	0.07	0.07	1	0.11	0.22	0.03	0.12	0.11	0.26	0.06	0.11	0	-0.01	0.01	0.1	-0.04	0	0.03
Dessert &Bakery	0.03	0.05	0.11	1	0.11	0.03	0.05	0.07	0.13	0.02	0.09	0	-0.01	0	0.05	-0.02	0	0.01
Fruits	0.11	0.1	0.22	0.11	1	0.05	0.2	0.13	0.25	0.04	0.13	-0.01	-0.03	-0.01	0.18	-0.08	-0.01	0.04
Herbs&Spices	0.01	0.02	0.03	0.03	0.05	1	0.02	0.02	0.03	0.01	0.03	0	-0.01	-0.01	0.05	-0.03	0.01	0.04
Menthol&Mint	0.11	0.1	0.12	0.05	0.2	0.02	1	0.06	0.11	0.06	0.18	-0.01	-0.01	-0.01	0.11	-0.07	0	0
Nutty	0.04	0.06	0.11	0.07	0.13	0.02	0.06	1	0.28	0.03	0.11	0	-0.01	0	0.07	-0.04	0	0
Cream	0.09	0.09	0.26	0.13	0.25	0.03	0.11	0.28	1	0.04	0.17	0	-0.02	0.01	0.14	-0.06	-0.01	0.02
Tobacco	0.01	0.02	0.06	0.02	0.04	0.01	0.06	0.03	0.04	1	0.03	0	-0.01	0	0.01	-0.02	0	0
Chocolate	0.1	0.12	0.11	0.09	0.13	0.03	0.18	0.11	0.17	0.03	1	0	-0.01	-0.01	0.08	-0.04	0	0
Promotion	0	-0.01	0	0	-0.01	0	-0.01	0	0	0	0	1	0.04	0.1	-0.14	0.16	0.04	0
#Post	-0.01	-0.01	-0.01	-0.01	-0.03	-0.01	-0.01	-0.01	-0.02	-0.01	-0.01	0.04	1	0.19	-0.12	-0.1	0.05	-0.03

Avg_share	0	-0.02	0.01	0	-0.01	-0.01	-0.01	0	0.01	0	-0.01	0.1	0.19	1	-0.04	-0.03	0.01	-0.03
Topic 1	0.05	0.05	0.1	0.05	0.18	0.05	0.11	0.07	0.14	0.01	0.08	-0.14	-0.12	-0.04	1	-0.52	-0.05	-0.07
Topic 2	-0.02	-0.02	-0.04	-0.02	-0.08	-0.03	-0.07	-0.04	-0.06	-0.02	-0.04	0.16	-0.1	-0.03	-0.52	1	0.01	0.21
URLmention	-0.01	0	0	0	-0.01	0.01	0	0	-0.01	0	0	0.04	0.05	0.01	-0.05	0.01	1	0.07
Hashtag	0	0	0.03	0.01	0.04	0.04	0	0	0.02	0	0	0	-0.03	-0.03	-0.07	0.21	0.07	1

The result shows that the largest Pearson correlation coefficient is 0.28 except the correlation between topic 1 and topic 2. For the flavor-related variables, the collinearity problem is not serious in our regression model.
